# Perceived greenness at home and at university are independently associated with mental health

**DOI:** 10.1186/s12889-020-8412-7

**Published:** 2020-05-28

**Authors:** Alexander K. F. Loder, A. R. Schwerdtfeger, Mireille N. M. van Poppel

**Affiliations:** 1 Institute of Sport Science, University of Graz, Graz, Austria; 2 Staff Department Quality Management, University of Music and Performing Arts Graz, Maiffredygasse 12b/I, 8010 Graz, Austria; 3 Institute of Psychology, University of Graz, Graz, Austria

**Keywords:** Neighborhood greenness, Public health, Mental health, Green space, Built environment, Austria

## Abstract

**Background:**

Previous studies reported positive associations between perceived neighborhood greenness and mental health. There has been a focus on perceived neighborhood greenness at people’s home environment or in general, but data are lacking on greenness at working places or other locations where they actually spend most of their time during their day.

**Methods:**

This study investigated the perceived greenness of college students’ home and study environments and its relation to mental health. An online survey collected data from 601 participants with a mean age of 24 years, living in or around and studying in the city of Graz, Austria. The perceived greenness at home and at university was assessed using questions on quality of and access to green space; mental health was measured with the WHO-5 well-being index. Uni- and multivariate regression analyses were used to analyze the data.

**Results:**

The analyses revealed positive associations between perceived greenness at home and mental health as well as perceived greenness at university and mental health. This adds more evidence to the existing literature that perceiving the environment as green is positively related to better mental health.

**Conclusions:**

Future research will have to incorporate objective greenness measures as a means of controlling for the reliability of the measurements and investigate the effects of different environments people are exposed to over the course of a day.

## Background

As long as human beings roamed the earth, nature and greenness were omnipresent parts of life. With human evolution came industrialization, advancing technology and sedentary behavior. From an evolutionary perspective, these general advancements, as well as grey colored facades, industrial areas and man-made pollution came to existence just about a blink of an eye ago. According to the biophilia-hypothesis, humans have developed and maintained an affinity for nature throughout their evolution [1, 2], which makes positive outcomes from exposure to green spaces on the human body and mind stand to reason. Correspondingly, “greenness” is linked to restorative effects on cognition and stress [3, 4], meaning that green space improves mental recovery after stress and reduces factors associated with decreased well-being.

### Neighborhood greenness and mental health

The presence and quality of nature as well as access to it, measured by vegetation indices or subjective perceptions, is referred to as neighborhood greenness. It is associated with better mental health outcomes such as a reduced risk of stress, propensity to psychiatric morbidity, psychological distress, depressive symptoms, clinical anxiety, depression and reduced need for mood disorder treatment in adults [5–13]. Despite this general evidence, a systematic review of the current literature on neighborhood greenness and mental health identified a lack of research in specific settings or for subgroups [14]. Additionally, the reported outcomes may not exclusively be limited to objective greenness measures, since there is research showing that perceiving the neighborhood as highly green is accompanied by better mental health outcomes compared to perceiving it less green [15]. Objective greenness measures were not included in this last study; thus the link between objective and perceived greenness could not be assessed.

Two main mechanisms have been proposed. First, there seems to be a preference for natural over built environments among humans, even when only photographs are presented (e.g. [16]). Accordingly, it was theorized that just having a view of nature is enough to improve affective restoration to a greater extent than built environments [17], meaning a reduction in the likelihood of emotional impulsive behavior and a reduced risk for mental illnesses. In line with this, a residents’ immediate home-environment was associated with higher satisfaction and well-being scores when there were natural elements in the view from their windows [18]. This mechanism can be defined as greenness influencing humans through a mere visual exposure to greenness.

A second hypothesized mechanism is that one has to be physically exposed to green space so that it can have an influence. This means that positive effects on human health can only be obtained once individuals move or stay in natural environments over a given timeframe (e.g. [1, 2, 19]). Most evidence for this hypothesis comes from cross-sectional studies that compared residents with high access to green in their neighborhood with residents with low access to green [14]. Benefits on mental health and health in general from being physically present in a green space can be derived from a host of variables. Neighborhood greenness provides opportunities for being physically active, e.g. by increasing walking for recreation, or by promoting social contacts, e.g. through increased interaction with other people [20, 21].

Most previous studies have assessed the relationship between green in the living environment and mental health. For instance, research focused on the amount of green around people’s residence or in the neighborhood, having a garden, the presence / number of green spaces within a certain distance or area, or the distance to the nearest green space (see: [14]). However, people do not only spend time at home during the day. Many adults spend much of their time at work and many students at university or other educational facilities. The latter subgroup is known to have a high potential to suffer from stressors, which can be created by moving out from home in conjunction with academic, social and financial pressure [22]. Similar to the findings related to greenness at home, we propose that green around university campuses has similar effects on mental health. This gets support from a study finding that students at universities with higher perceived greenness of a university campus are more likely to self-report higher quality of life compared to students with lower perceived greenness [23]. It is, however, not expected that direct greenness exposure (being outdoors in the green environment for prolonged times) is the major driver of mental health benefits in this case. Home-environments are often chosen based on their natural surroundings and the “dose” of nature may depend on the actual time people use the green space. In line with this, there is support from a study that reported a decline in green space use with increasing distance from the home-environment [24]. University-environments, where people are not as likely to spend much of their free time as at home, are usually not chosen with green space as criteria in mind. We suppose that possible positive effects from green space around students’ campuses will be based more on the first mechanism, which is concerned with mental health outcomes by looking at nature. There is a more limited opportunity to direct exposure to it, e.g. when walking around on the campus during classes, compared to home environments. Furthermore, the relation of green around the study environment with mental health might be less influenced by social economic status or a person’s own preference for their living environment.

This study extends the current body of evidence by assessing the associations between perceived greenness and mental health and, specifically, includes the perceived greenness both at home and at university.

## Methods

As part of a larger online-survey data collection was conducted between October and December 2017. This survey obtained data of self-reported neighborhood greenness and different outcome variables such as mental health, means of transport and body mass index as well as the approximate location where people lived and worked / studied. Its purpose was to identify associations between greenness and health, and assess possible mediating factors, dependent on different locations. Previously, we reported on the association of perceived greenness at home and at university with BMI, sedentary time and physical activity behavior [25]. The study at hand investigated the association of perceived greenness with mental health. Students with both residence in and around and study environment in the city of Graz (Austria, capital of Styria) were selected. The city of Graz has 300,000 residents, spans 128 km^2^ and is predominantly known for its universities. The majority of the sample lived, studied or spent the most time during the day in the university area of Graz, which is located at the edge of he city center, surrounded by green space and park area. The ethics committee of the University of Graz has approved this study (GZ 39/58/63 ex 2016/17).

### Power analysis

Based on literature, we generally expected small effect sizes [26]. Sample size and statistical power were calculated using G*Power [27]. Based on pooled effect sizes from a literature review [26], effects of around .20 were considered a meaningful assumption prior to data collection for calculating minimum sample size. For multivariate regression models with five predictors and a α-level of .05 a sample size of *n* = 100 would be needed to detect this effect size. As data collection was accomplished via an online-survey, accumulating a larger sample for detecting possible smaller effects was feasible.

### Study population and data collection

Methods of data collection have been previously described [25]. In short, participants of the online survey were recruited via social media and university-related mail distribution lists. It was aimed to get a diverse sample of workers and students with a representative range of home, work and study environments in and around the city of Graz. A total of 758 respondents finished filling in the questionnaires, and after exclusion of those who did not provide informed consent (*n* = 115), and who were not “college student” (*n* = 42), the sample in the analysis consisted of 601 students.

### Study variables and questionnaires

#### Perceived greenness measures

As no standardized questionnaire for perceived neighborhood greenness were available, a reduced set of questions from a previously published questionnaire was used [28]. Six questions centered on the quality of green space and the access to it [28, 29]. The participants had to answer each question two times, first for the environment at home and then for their study environment / workplace (or the place where they used to spend the most time during the day), adding up to 12 questions overall.

The home- and the study-environments were only specified by referring to the greenness “at home” in one set of questions and the greenness “at the study- / working place or the place where they spend most of their time during the day (e.g. school, university)” in the other set. There was no detailed explanation of these locations. We used the mean scores of each of the measures for perceived greenness at home and at the study environment as two distinct variables that were transformed into percentages. A third overall score was calculated from the average of these variables, as follows: (perceived greenness at home in percent + perceived greenness at university in percent) / 2. This variable, as an estimate of participants’ perception of nature for the majority of the day, served as a general measure for perceived greenness over the day. As other studies are often lacking a distinction between greenness in different locations, we included this score for comparability purposes [14]. All perceived greenness measures, i.e. at home, at university and overall, ranged from 0 – very bad to 100 – very good.

#### Mental health: WHO (five) well-being-index

Mental health was assessed with the German version of the WHO (Five) Well-Being-Index (World Health Organization), which concentrates on the last 2 weeks of wellbeing [30, 31]. On a scale from 5 to 0 (5 “all of the time”, 4 “Most of the time”, 3 “More than half of the time”, 2 “Less than half of the time”, 1 “Some of the time”, 0 “at no time”) the following five questions make up the questionnaire:

- I have felt cheerful and in good spirits
- I have felt calm and relaxed
- I have felt active and vigorous
- I woke up feeling fresh and rested
- My daily life has been filled with things that interest me

A literature review on the psychometric properties of this questionnaire including more than 200 studies summarized that the WHO (Five) Well-Being-Index has high clinimetric validity, high construct validity (the items represent a unidimensional scale) and high predictive validity. It has a high sensitivity and a moderate to high specificity for major depression. It is also applicable to other settings as it was used for a variety of scenarios such as wellbeing in occupational health-settings, for instance [32].

We decided not to follow the original scoring principle, e.g. described in Topp, Østergaard, Søndergaard and Bech [32]. This would have required summing up the raw scores and multiplying the result by four to get a measure between 0 and 100. Instead, we calculated the mean value of the raw scores, which was then transformed into a percentage (from 0 “at no time” to 100 “all of the time”). This allows for including datasets with missing values, which is not possible for sum scores, and was used because the questionnaire had no mandatory questions for the sake of participants’ compliance. The calculation was done as follows: 100 / maximum score [5] * mean of item scores. This measure was used as the dependent variable for measuring mental health.

As a measure of mental health this questionnaire is used as an indicator for depression in the literature with different cutoff-scores resulting in different sensitivity and specificity scores (see: [32]). The majority of the studies reviewed by Topp, Østergaard, Søndergaard and Bech [32] that investigated mental health used a cutoff score of ≤50 of the WHO (Five) Well-Being-Index, when using the sum scores times four, to assign a screening diagnosis of depression [30, 31]. Based on this evidence, we defined values ≤50, i.e. half of the maximum value, as indication of low mental health.

### Statistical analysis

Correlational analyses were conducted via Pearson correlational analyses. These analyses were used to check how strongly the perceived greenness scores of the home-environment were linked to the scores of perceived greenness at university.

The associations between perceived neighborhood greenness and mental health were analyzed by using single- and multivariate linear regression models. Both perceived neighborhood greenness for the home- and the study-environment were included as exposure measures in the analyses. Potential confounders were identified based on literature (gender, age, income) (e.g. [33–36]), and entered in the models simultaneously. Age was used as a continuous variable and income (less than 1000 €, 1001 € to 3000 €, more than 3001 €) as well as gender were dummy-coded and used as categorical variables. The reference category in this model for gender was “women” and for income “less than 1,000 €”. Missing values in confounders were handled using a multivariate regression model with multiple imputation. Random seed for numbers in the imputation process was set to 1 and the number of imputations to 100 [34]. All Analyses were conducted using IBM SPSS 25®.

## Results

### Sample

A total of 601 participants made up the sample. 465 (77%) of these participants were female at a mean age of 24 (*SD* = 7) years. Detailed sample characteristics are shown in Table 1.

**Table 1:** Sample characteristics

Variable	Value(s)
	*N* = 601
Gender	465 (77%) female
	125 (21%) male
	11 (3%) missing
Marital status	20 (3%) married
	258 (43%) in a partnership
	281 (47%) single
	42 (7%) missing
Income per month	374 (62%) less than 1000 €
	88 (15%) between 1001 € and 3000 €
	10 (2%) more than 3001 €
	129 (22%) missing
Living in the city of Graz	450 (75%)
Studying in the city of Graz	543 (90%)
Age in years, *M* (*SD*)	24 (7)
Mental health, *M* (*SD*)	57.36% (18.84%) 157 (26%) indications of low mental health (score ≤ 50%)
Perceived greenness at home, *M* (*SD*)	79.86% (16.40%)
Perceived greenness at work, *M* (*SD*)	69.78% *(*19.45%)
Perceived greenness overall, *M* (*SD*)	74.86% *(*14.14%)

### Testing for assumptions of multivariate regression analysis for confounder testing

Collinearity checks were conducted using Pearson-correlations for the combined data after multiple imputation. For the results see Table 2. The highest correlation coefficient obtained was a moderate effect between age and the dummy-coded income-category “1,001 € - 3,000 €” (*r* = .47, *p* < .001). In the original dataset (without dummy-coding) the tolerance statistics and variance inflation factor did not indicate that collinearity was of concern, as shown in Table S1. Furthermore, the assumption of independent errors (*Durbin-Watson value* = 2.08) met the assumption of independent errors and Cook’s distance values were lower than a value of 1. This indicates hardly an unduly influence in the regression model of individual cases. Scatterplots of standardized residuals (original dataset) and for the datasets of the imputation show fulfilled assumptions of homogeneity of variance and linearity. The errors should be approximately normally distributed as assessed by visual review of Normal P-P plots of standardized residuals, which were showing almost all dots on and close to the line. These results were obtained for both the original dataset and the imputed datasets.

**Table 2:** Correlation matrix of possible confounders in the combined datasets after multiple imputation

	WHO-5	Perceived Greenness at home	Perceived Greenness at university	Gender (men)	Age	Income 1001-3000€
WHO-5						
Perceived Greenness at home	*r* = .15 *p* < .001					
Perceived Greenness at work	*r* = .18 *p* < .001	*r* = .25 *p* < .001				
Gender (men)	*r* = .11 *p* = .015	*r* = .02 *p* = .375	*r* = .04 *p* = .235			
Age	*r* = −.01 *p* = .459	*r* = .06 *p* = .081	*r* = .01 *p* = .448	*r* = .14 *p* = .001		
Income 1001-3000€	*r* = .09 *p* = .041	*r* = .01 *p* = .414	*r* = .001 *p* = .490	*r* = .07 *p* = .070	*r* = .47 *p* < .001	
Income > 3000 €	*r* = .08 *p* = .056	*r* = .07 *p* = .078	*r* = −.02 *p* = .375	*r* = .03 *p* = .291	*r* = .22 *p* < .001	*r* = −.07 *p* = .063

### Perceived neighborhood greenness at home and at university

The scores of perceived greenness of the home-environment (*M* = 79.86%, *SD* = 16.40%) as well as the study-environment (*M* = 69.78%, *SD* = 19.45%) were generally high. Furthermore, a third measure – overall perceived-greenness – was derived from the mean scores of the previous two variables of perceived greenness (*M* = 74.86%, *SD* = 14.14%). A significant but moderate correlation between perceived greenness at home and at university was obtained from Pearson-correlations (*r* = .24, *p* < .001).

### Perceived neighborhood greenness and mental health

The results of the linear regression analyses of perceived greenness and mental health can be found in Table 3 (for an extended version see Table S2). Perceived greenness at home, at university and overall were all significantly associated with mental health. Univariate associations with mental health were similar for greenness at home and at university. When adding greenness of both locations into the model simultaneously, both remained significantly associated with mental health. Adjustment for gender, age and income did not change the strength of the associations.

**Table 3:** Linear regression models with mental health as outcome and perceived greenness as well as possible confounders as indicators

	*R*^2^	*b*	*p*	95% CI *b*
At home	.02	0.17	.001	0.07–0.27
At university	.03	0.17	< .001	0.09–0.26
Overall	.04	0.28	< .001	0.16–0.39
Multivariate				
At home and at work	.04		< .001	
At home		0.13	.020	0.02–0.23
At university		0.14	.001	0.06–0.24
Multivariate adjusted				
With confounders			< .001	
At home		0.14	.022	0.02–0.26
At university		0.17	.001	0.07–0.26
Gender (men)		4.98	.031	0.46–9.50
Age		−0.32	.040	−0.62 – −0.02
Income (1001-3000€)		6.81	.012	1.49–12.13
Income (> 3000€)		13.78	.033	1.08–26.48

## Discussion

### Main findings

In line with the current literature on perceived greenness and mental health [14], this study found positive associations between these factors. This means that perceiving the neighborhood at home as highly green is linked to better mental health than perceiving it less green in students.

However, most studies only addressed neighborhood greenness in unspecified locations, in general or in the home-environment [14]. Since students do not only spend time at home during the day, focusing on this location only might be too limited. Including the perceived greenness of the study-environment in this study revealed positive associations between the perceived greenness at university and mental health scores, independent from greenness at home.

### Perceived greenness at home and at university

We obtained relatively high perceived greenness measures for both the home environment and at university. This is likely due to the green space surrounding the university area, i.e. the small distance to parks, and the generally green campus of the University of Graz. It was controlled for a possible subjective bias. We found that only some people perceived the environment greener than others. There was a moderate correlation between perceived greenness at home and at university, which indicates only an negligible influence on the outcomes of the analyses due to “general high and low greenness-perceivers”.

The greatest part of the prevailing literature on green space and mental health did not incorporate greenness in distinct locations (e.g. see: 14). Thus, including the perceived greenness measures at students’ living environments and study environments reveals new insights into how nature affects mental health. To further understand the role of green at university, more information on how this green is used and through which mechanism it acts on mental health (visual exposure only, being more physically active in green or having more social interactions) needs to be gathered. Future research will have to address this issue in closer detail with study designs allowing for a clear differentiation between direct greenness exposure and viewing green, which was not possible in this study.

In addition, our results indicate that green at different locations may have an independent effect on mental health. In line with our results for the overall score of perceived greenness, this might possibly indicate that there is a dose-response relationship and that the more people are exposed to green over the day, the better their mental health is. Our findings indicate that at study environments, efforts should be made to enable students (and workers) to look outside and be outside in a green environment. More specific study designs will be needed to accumulate more evidence for this assumption in order to be able to generalize it to other subgroups beyond the university context.

### Confounding variables

Our results showed that age is not associated with mental health, possibly due to the limited age range of our population. Income was positively associated with mental health, congruent with the evidence from a study observing a higher prevalence of mental disorders among women living in poverty than women in the general population [33] and another study reporting higher odds ratios for mental disorders among individuals with low income compared to individuals with higher income [35]. Low socio-economic-status neighborhoods are also associated with low mental health [37]. In addition, we found better mental health in men compared to women, similar to one study that reported a positive relationship between mental health and greenness within wards for men, but not for women [38]. However, adjusting for age, gender or income did not change the association between greenness and mental health in our study.

### Strengths and limitations

An important point to discuss when interpreting the reported results is sample size in the context of possible influences on the statistical significances observed. Congruent with the findings of this study, previous research also predominantly reports low to moderate effect sizes in regards of neighborhood greenness and mental health, i.e. focusing on sadness / depression (for a review see: 27). Since most of the effect sizes are small, the “clinical” relevance of the results might be limited. The exclusive student sample could be one reason for the small effect sizes, since the perceived greenness levels at the university’s environment might be relatively homogeneous and the variance of the scores of the greenness variables could be limited. We suppose that this is possible as almost all participants lived near and studied in the same places. Therefore it stands to reason that larger effects can be obtained in more diverse samples.

In respect of the small effect sizes and given the actual sample of 601 people, multivariate regression analyses with five predictors equate a power level of *R*^2^ = .77 and univariate models a power level of *R*^2^ = .88. Relying on statistical recommendations, it should be noted that these values ensure a good to very good power for the research that was conducted [39, 40].

People are rather choosing highly-green residential environments in cities, i.e. more favorable ones, when they can afford to live there [41]. Therefore, it is plausible that socioeconomic status and income are influencing the outcomes of research not controlling for these dimensions. This student sample is also. Due to homogenous makeup of the sample in respect of age, socio economic status, income and education, most participants had high education levels, while being low in income, i.e. lower than 1000 € per month. Therefore socioeconomic factors as confounders were unlikely in this sample. However, this also limits the sample’s representativeness for all adult residents in the city of Graz.

Standardized and validated questionnaires for perceived greenness in the same place or distinct locations are currently not available. This is the reason why it is not clear whether the questions to measure perceived greenness were reliably assessing the desired outcomes. The subset of six questions we took from the previously existing questionnaire of the PHENOTYPE project (see: 29) is similar to the items from the Neighborhood Environment Walkability Scale [42]. Another study addressing similar objectives regarding perceived greenness used this specific questionnaire [15]. This scale contains items concerned with access to park or nature reserve, access to bicycle and walking paths, presence of greenery and presence of tree cover or canopy along footpaths as well as presence of pleasant natural features [42]. This increases the comparability of the perceived greenness measures of other studies, although it does not directly influence the reliability and validity of the set of questions used in the study at hand.

As most previous studies, the study at hand is based on a cross-sectional study design. Therefore the causal direction of the results is not clear making a causal relationship between the greenness and mental health measures of this study impossible to establish. Associations found might be confounded by the discussed variables as well as by other, partially unknown, differences between those living in green areas and those that do not. Although the literature predominantly assumes that neighborhood greenness is affecting mental health [14], the relationship between these factors could act in a reverse manner. There is evidence that green environments are linked to higher levels of self-reported happiness than less green environments [43]. On the contrary, poor mental health, for instance in case of major depression, is able to negatively affect people’s mood [44]. Thus, it seems reasonable that mental health might also be affecting the perception of the neighborhood and the quality of green space. However, we assume that these factors are related to different sources of variance and based on the current body of evidence [14] a (strong) directional association of mental health affecting perceived greenness is rather unlikely in our sample.

### Prospect

Based on the results of the current study, future research could have a closer look at the association between both perceived and objective greenness and mental health in the context of different environments, such as at home, at university and at work. It is likely that positive associations between mental health and objective greenness will be obtained given a similar sample as in this study. There is similar evidence from another study focusing on objective greenness measures in the home environments of an exclusive student sample. Positive associations between greenness in students’ home environment and mental health were found [45]. In combination with the higher ratings of quality of life when perceived greenness is high at university [23] we assume that it is likely that future studies on objective greenness in different places will show similar results as our study.

Moreover, more specific study designs should also have a look at the direction and causation of this relationship, since it is not clear if the associations are of uni- or bidirectional nature. Additionally, the dose-response relationship between greenness and its effects on mental health might need further examining, meaning how much exposure is necessary to improve mental health to which extent. Another leverage point for further research should be the development of standardized measures for perceived greenness. In line with this, including measures for objective greenness in a study design and controlling for them could possibly increase the reliability of the results for perceived greenness. As there is evidence in regards of a lack of agreement between perceived and objective measures [46], this can be done by comparing and matching both indicators.

## Conclusions

This study found positive associations between perceived greenness and mental health both in the home and study environment. This indicates that more attention should be paid to developing green university campuses, in addition to green living areas. Future studies are needed in older and more general (working) populations and in other geographic locations.

## Supplementary information

**Additional file 1:** Collinearity statistic of the original dataset in multivariate regression analysis for confounder testing.

**Additional file 2:** Linear regression models with mental health as outcome and perceived greenness as well as possible confounders as indicators.

## Data Availability

The datasets used and / or analyzed during the current study are available from the corresponding author on reasonable request.

## Abbreviations

PHENOTYPE: Positive health effects on the natural outdoor environment in typical populations of different regions in Europe
WHO: World Health Organization
