# Supplementary material for: Perceived greenness at home and at university are independently associated with mental health
Source: BMC Public Health. 2020 May 28;20:802. doi: 10.1186/s12889-020-8412-7 (PMC7254725; doi:10.1186/s12889-020-8412-7)
Supplement: Supplementary file 1 — Additional file 1: Collinearity statistic of the original dataset in multivariate regression analysis for confounder testing. [file 12889_2020_8412_MOESM1_ESM.docx]

Table S1

*Collinearity statistic of the original dataset in multivariate regression analysis for confounder testing.*

| **Variable** | **Tolerance** | **Variance Inflation Factor** |
| --- | --- | --- |
| Perceived Greenness at Home | .91 | 1.10 |
| Perceived Greenness at University | .92 | 1.09 |
| Gender | .98 | 1.02 |
| Age | .71 | 1.42 |
| Income | .72 | 1.39 |
