# Supplementary material for: Perceived greenness at home and at university are independently associated with mental health
Source: BMC Public Health. 2020 May 28;20:802. doi: 10.1186/s12889-020-8412-7 (PMC7254725; doi:10.1186/s12889-020-8412-7)
Supplement: Supplementary file 2 — Additional file 2: Linear regression models with mental health as outcome and perceived greenness as well as possible confounders as indicators. [file 12889_2020_8412_MOESM2_ESM.docx]

Table S2 (extended from Table 2)

*Linear regression models with mental health as outcome and perceived greenness as well as possible confounders as indicators.*

|  | *R*^2^ | *F* | *df* | *p* | *b* | *t* | *df* | 95% CI *b* |
| --- | --- | --- | --- | --- | --- | --- | --- | --- |
| At home | .02 | 10.74 | 1, 471 | .001 | 0.17 | 3.28 | 471 | 0.07 – 0.27 |
| At university | .03 | 15.41 | 1, 468 | < .001 | 0.17 | 3.93 | 468 | 0.09 – 0.26 |
| Overall | .04 | 21.35 | 1, 471 | < .001 | 0.28 | 4.62 | 471 | 0.16 – 0.39 |
| Multivariate | | | | | | | | |
| At home and at university | .04 | 10.49 | 2, 467 | < .001 |  |  |  |  |
| At home |  |  |  | .020 | 0.13 | 2.33 | 467 | 0.02 – 0.23 |
| At university |  |  |  | .001 | 0.14 | 3.24 | 467 | 0.06 – 0.24 |
| Multivariate adjusted | | | | | | | | |
| With confounders |  |  |  | < .001 |  |  |  |  |
| At home |  |  |  | .022 | 0.14 | 2.29 | 459 | 0.02 – 0.26 |
| At university |  |  |  | .001 | 0.17 | 3.35 | 459 | 0.07 – 0.26 |
| Gender (men) |  |  |  | .031 | 4.98 | 2.16 | 459 | 0.46 – 9.50 |
| Age |  |  |  | .040 | -0.32 | -2.06 | 459 | -0.62 – -0.02 |
| Income (1,001-3,000€) |  |  |  | .012 | 6.81 | 2.51 | 459 | 1.49 – 12.13 |
| Income  (> 3,000 €) |  |  |  | .033 | 13.78 | 2.13 | 595 | 1.08 – 26.48 |
